# Super special relativity

**DOI:** 10.3389/fncom.2025.1597914

**Published:** 2025-08-13

**Authors:** Nicholas Jordan Wagter

**Affiliations:** Department of Medical Biophysics, Western University, London, ON, Canada

**Keywords:** time, perception, computation, information processing, framework

## Abstract

This paper proposes a new theoretical framework for understanding time perception centered on information processing in the brain. We introduce the concept of “perceptual time” as distinct from inertial clock time and develop a model relating perceptual time experience to the brain’s computational capacity and information processing rate. This framework explains phenomena like time dilation and compression during intense experiences in terms of neural information processing, bridging perceptual time with physical theories of time.

## 1 Introduction

Our experience of time is fundamental to consciousness, yet often diverges from inertial clock time. Moments can stretch or compress depending on our mental state and environmental conditions. While Einstein’s theory of relativity established that inertial time measured by a standard clock in a non-accelerating (inertial) reference frame is relative to the observer’s position and motion in physical space–time, we propose that inertial time is also relative to the observer’s internal frame of reference, which is their neural information processing capacity.

This paper introduces a theoretical framework for “perceptual time” based on information theory and computational neuroscience. Perceptual time experience emerges from the brain’s information processing and can be modeled as a function of neural computational capacity and information processing rate. This relationship provides a quantitative approach to characterizing phenomena such as perceptual time compression during intense experiences or perceptual time dilation during flow states.

The proposed model holds significant potential for real-world applications. Understanding perceptual time variations can help us understand and potentially aid in the development of therapeutics for ailments that affect time perception, such as Alzheimer’s and Parkinson’s disease. Additionally, time perception plays a crucial role in productivity, as the ability to perceive time impacts workload estimation. Understanding the brain’s true capacity to perform tasks can help set realistic workplace expectations and optimize business operations.

Moreover, the Perceptual Time Model will also have broad implications with respect to brain-computer interfaces (BCIs) and artificial intelligence (AI) systems. Future iterations of brain-computer interfaces could leverage the model to modulate an individual’s perception of time, potentially enhancing focus or mitigating the perception of time pressure in high-stress scenarios by compressing perceptual time. AI systems, on the other hand, could adopt perceptual time as a more adaptive metric for measuring a system’s evolution, allowing for a granular understanding of task prioritization and decision-making in dynamic environments. However, further empirical research is necessary to validate the model and develop practical methods for measuring the key cognitive variables that influence perceptual time.

## 2 Background

The perception of time is a foundational aspect of conscious experience, with time being inescapable and pervasive in our individual experiences (Callender, 2017; James, 1890). Time holds a special status as it is the only property that persists in both the content and structure of our experiences, making it a strong candidate for a minimal unifying model of consciousness (Dainton, 2008; Phillips, 2010; Chuard, 2011; Windt, 2015). Understanding how the brain processes temporal information and gives rise to our perception of time is complex, given the intricate network of billions of neurons and trillions of synaptic connections. Given the complexity, extensive research has been conducted, some consistent and some conflicting, but the challenge remains in developing a comprehensive framework.

### 2.1 Existing computational models of time perception

Various theories, such as the internal clock theory (Church, 1984) and the behavioral theory of timing (Killeen and Fetterman, 1988), have sought to explain the neural mechanisms underlying time perception. Computational and robotic models have been developed to study these theories, including the pacemaker-accumulator model (Simen et al., 2013) and the memory decay model (Addyman et al., 2011). The pacemaker-accumulator model provides a framework for how time and duration are perceived, insisting that a mechanism in the brain (the pacemaker) sends pulses to an accumulator in the brain that counts the pulses to deduce time. The theory further suggests the presence of a “switch” between the pacemaker and accumulator that activates depending on whether the brain is actively paying attention to time (Simen et al., 2013). In contrast, the memory decay model of time perception posits that our temporal judgments are influenced by the gradual weakening of memory traces. As retention intervals increase, there is a progressive strengthening of central bias, where individuals place greater weight on expected rather than actual duration distributions when perceiving incoming sensory stimuli (Addyman et al., 2011). Review papers like Basgol et al. (2021) have synthesized these works, but there remains a need for a comprehensive framework that exploits temporal information to govern agentic behavior.

In addressing the computational mechanisms underlying temporal processing, the State-Dependent Network (SDN) model (Karmarkar and Buonomano, 2007) demonstrates that temporal information can be encoded through intrinsic neural dynamics via short-term synaptic plasticity and GABA_B-mediated inhibition, rather than dedicated timing mechanisms. This context-dependent framework, wherein temporal encoding varies with the network’s initial state, provides a mechanistic explanation for how internal mental states influence time perception. However, the model’s severe temporal limitations (<500 ms), are validation restricted to simple discrimination tasks, and parameter sensitivity undermines its explanatory power, particularly as its inability to account for longer durations necessitates hybrid approaches that reintroduce the dedicated timing mechanisms it sought to replace.

### 2.2 Neural correlates and behavioral evidence for modeling time perception

In the pursuit of a unified model of time perception, numerous studies have revealed striking parallels between numerical and temporal processing, as evidenced by behavioral, neural, and clinical data (Dormal et al., 2006; Feigenson, 2007; Provasi et al., 2011; Dormal and Pesenti, 2012, 2013; Vicario et al., 2013). These parallels, supported by behavioral, neural, and clinical evidence, suggest shared underlying mechanisms that could form the foundation of such a model. Behavioral data indicates that both numerical and temporal judgments rely on Weber’s Law, with discrimination being more straightforward when quantities differ by a larger ratio (Stevens, 1957). Weber’s law states that the just-noticeable difference between two stimuli is proportional to the magnitude of the stimuli (Fechner, 1966). For instance, rats and infants have demonstrated the ability to generalize rules learned in one area, such as time or numbers, to the other (Meck and Church, 1983; de Hevia et al., 2012). However, in the case of infants, similar developmental trajectories are observed during infancy, but divergence emerges in childhood (Odic, 2017).

This highlights the need for a plausible foundational model of perceptual time that can accommodate these developmental deviations. While our current framework is not age-parameterized, it can account for these effects by attributing developmental changes to evolving neural architecture, specifically, changes in the effective number of processors, efficiency, and power consumption. As cortical networks mature and cognitive functions like attention and working memory strengthen, the brain’s capacity to process temporal information becomes more differentiated. Future empirical work could use this framework to map developmental trends in time estimation to underlying neural computation parameters.

Additionally, neuroimaging studies have shown intraparietal sulcus (IPS) activation during both numerical and temporal processing in adults (Dormal et al., 2012; Skagerlund et al., 2016; Hayashi et al., 2013), suggesting that the comorbid deficits in quantity processing observed in clinical disorders like Turner syndrome—linked to spatial reasoning and working memory impairments—may arise from disruptions in the neural mechanisms supporting these overlapping cognitive functions (Vicario et al., 2013). The overall consensus is that the IPS is implicated more strongly in numerical than temporal processing (Rammsayer and Classen, 1997; Nenadic et al., 2003; Mattel and Meck, 2004; Koch et al., 2009), which led to the proposal of a standard magnitude system responsible for processing both time and numbers (Meck and Church, 1983; Walsh, 2003; Cantlon et al., 2009).

Given the findings from IPS and laboratory studies, the correlation between temporal and numerical processing was previously a key foundational component of time perception. However, inconsistencies in subsequent research have ultimately discredited its inclusion in models (Baker et al., 2013; Young and Cordes, 2013; Odic, 2017), highlighting the need for a plausible foundational model for perceptual time that rectifies inconsistencies in existing research.

As research advances in the fields of biology and neuroscience, it is increasingly apparent that the brain’s mechanisms for perceiving time are highly complex, posing significant challenges for developing a comprehensive understanding of these processes. A simplified theoretical model that abstracts the complexities of neural computation and focuses on critical parameters governing the brain’s information processing capacity has the potential to elucidate how computational limitations lead to perceptual time distortions. By building upon fundamental principles of information processing, energy consumption, and physical constraints, such a model can capture the essential aspects of perceptual time.

## 3 Theoretical framework

### 3.1 Introduction

In this paper we propose a simplified theoretical model, the Perceptual Model of Time, to explore how the brain’s computational limitations might lead to perceptual time distortions. This model abstracts the complexities of neural computations and focuses on key physical parameters that govern the brain’s information processing capacity.

### 3.2 Fundamental principles and simplifying assumptions

Our model is built upon three fundamental principles:

1. Information Processing: The brain continually processes information from sensory inputs, internal signals, and cognitive activities. A study by Panagiotaropoulos et al. (2012) demonstrated this by showing that visual awareness is reflected in power modulations of high-frequency local field potentials in the lateral prefrontal cortex of macaque monkeys. This finding suggests that neuronal populations in this associated cortical area represent the content of conscious visual perception.
2. Energy Consumption: Information processing requires energy, with the brain consuming a significant portion of the body’s metabolic resources. Neuroimaging studies have shown activation of the IPS during both numerical and temporal processing in adults, indicating an apparent energy demand during these processes (Dormal et al., 2012; Skagerlund et al., 2016; Hayashi et al., 2013).
3. Physical Constraints: Physical factors such as neuronal transmission speeds, synaptic efficiency, and energy availability limit the brain’s information processing capacity. These constraints have been explored through computational and robotic models, including the pacemaker-accumulator model (Simen et al., 2013) and the memory decay model (Addyman et al., 2011).

By acknowledging these principles, we can construct a model that, while simple, captures the essential aspects of how computational limitations affect perceptual time. This approach builds upon the existing body of research synthesized in review papers like Basgol et al. (2021) while providing a novel framework that exploits temporal information to govern agentic behavior.

### 3.3 Integration with background models

While traditional frameworks such as the pacemaker-accumulator (Simen et al., 2013) and memory decay models (Addyman et al., 2011) focus on the internal generation of temporal signals or the degradation of memory traces over time, our approach shifts emphasis to the computational constraints that govern overall neural information processing capacity. Rather than relying solely on clock-like mechanisms or passive forgetting, our model incorporates fundamental physical and metabolic constraints—such as parallel processing capabilities, energy consumption, and efficiency factors—to provide a broader, capacity-limited perspective on how time perception emerges from the brain’s underlying information-processing architecture.

### 3.4 The basic model

We distinguish perceptual time (t’) from inertial time (t) and introduce the following key variables:

I: Total information processed by the brain (bits)

N: Number of processors (e.g., neurons or functional computing units)

P: Power consumption per processor (watts)

η: Efficiency factor (bits/joule)

T: Absolute temperature of the brain (kelvin)

Step 1: Define the maximum computational rate of the brain (R) (in bits/s) as:


(1) R=N×P×η

Equation 1 represents the total rate at which the brain can process information.

Step 2: Relate perceptual time to the total information processed (I) and the computational rate (R):


(2) $$t\prime =\frac{I}{R}=\frac{I}{N\times P\times \eta }$$

This implies that perceptual time is proportional to the total information processed divided by the processing capacity, aligning with our conceptual framework.

*Examples:*

In the context of perceptual time, the relationship between inertial time and perceptual time can be represented by various ratios.

A 1:1 ratio indicates that 1 s of perceptual time is equivalent to 1 s of inertial time, suggesting a perfect correspondence between the two. However, deviations from this ratio can occur, leading to either a compression or dilation of perceptual time.

When the ratio is 1:0.5, 1 s of perceptual time is equal to 0.5 s of inertial time, indicating a compression of perceptual time (moments feel longer or slower).

Conversely, a ratio of 1:2 represents a dilation of perceptual time, where 1 s of perceptual time is equal to 2 s of inertial time (moments feel shorter or faster).

#### 3.4.1 Analogies

##### 3.4.1.1 Velocity equation

Equation 2 is analogous to the familiar formula t=d/v (time equals distance divided by velocity). Both equations relate a quantity to the ratio of two other quantities: in t=d/v, time (t) is related to the ratio of distance (d) and velocity (v), while in Equation 2, perceptual time (t′) is related to the ratio of total information processed (I) and the computational rate (N×P×η). The structure of the equations is also similar, with the “perceived” quantity (perceptual time) being equal to the ratio of an “actual” quantity (distance or information) divided by another “actual” quantity (velocity or computational rate). This analogy helps to illustrate how the proposed equation relates perceptual time to information processing in the brain.

##### 3.4.1.2 Car age

Consider a car as an illustrative analogy. Its “inertial time” can be likened to the number of calendar years since its manufacture which is an objective measure independent of usage. In contrast, its “perceptual time” is more analogous to the car’s total accumulated mileage, which reflects the intensity and frequency of its operation. Thus, a vehicle that has existed for many years but has been driven minimally may exhibit characteristics suggesting relatively limited “aging” in operational terms. Conversely, a newer vehicle subjected to extensive and demanding use may demonstrate functional degradation that appears disproportionate to its chronological age. In this manner, inertial time corresponds to a simple temporal count, while perceptual time approximated by mileage, captures the depth and complexity of experiential wear.

##### 3.4.1.3 Interpretation

The basic model (Equation 2) provides a foundation for understanding how the brain’s computational limitations may influence perceptual time. The model reveals several critical implications regarding information processing, parallel processing, power consumption, cognitive load, and efficiency.

Firstly, perceptual time (t′) is directly proportional to the total information processed (I) by the brain, suggesting that as the brain processes more information within a given inertial time frame, the perceived time within that inertial time interval may decrease. This aligns with empirical studies indicating that increased cognitive load often leads to underestimation of inertial time. For instance, Castellotti et al. (2022) found that participants performing more difficult cognitive tasks tended to underestimate the duration, perceiving inertial time as passing more quickly, suggesting that while more information is processed, the allocation of attention to the task reduces the awareness of inertial time.

Secondly, the relationship between perceptual time and the number of processors (N) is inversely proportional, implying that a higher number of parallel processing units in the brain could compress perceptual time. This concept is supported by a study conducted by Sigman and Dehaene (2008), which examined brain mechanisms of serial and parallel processing during dual-task performance. The researchers found that certain brain networks, including bilateral posterior parietal cortex, premotor cortex, supplementary motor area, anterior insula, and cerebellum, were shared by both tasks during dual-task performance, suggesting parallel processing. This parallel processing capability allows the brain to distribute tasks and could lead to a compression of perceptual time, as multiple tasks are processed simultaneously.

Lastly, the efficiency factor (*η*) is inversely proportional to perceptual time, implying that individuals with more efficient neural computations may experience inertial time passing more slowly compared to those with less efficient processing. Factors such as attention, emotional state, and memory can affect processing efficiency. The study by Castellotti et al. (2022) demonstrates that cognitive and motor tasks interfere with time perception, suggesting that efficient allocation of cognitive resources influences how time is perceived. The efficiency factor (η) should be treated as a variable encompassing various cognitive and emotional influences on perceptual time, and future work should aim to quantify the impact of these factors on (η) and incorporate them into the model more explicitly.

These interpretations provide a conceptual framework for understanding how the brain’s computational capacity, parallel processing, power consumption, and efficiency may contribute to perceptual time distortions. The basic model suggests that variations in these parameters, either across individuals or within an individual over time, could lead to differences in perceived time. Advancing our understanding of their influence is valuable for several purposes, such as estimating mental capacity in the workplace, and developing therapeutics for conditions that affect time perception, such as Turner syndrome, Alzheimer’s disease, schizophrenia, and Parkinson’s disease.

However, it is important to recognize that this basic model is a simplified representation of the complex neural processes underlying time perception. The brain’s information processing is influenced by numerous factors, such as attention, emotion, memory, and sensory input, which are not explicitly captured in this model. Further refinements and extensions to the foundational model will be necessary to incorporate various additional factors to provide a more comprehensive understanding of perceptual time.

### 3.5 Incorporating Landauer’s Principle

Landauer’s Principle, proposed by Landauer (1961), states that any logically irreversible manipulation of information, such as the erasure of a bit, must be accompanied by a corresponding entropy increase in non-information-bearing degrees of freedom of the information-processing apparatus or its environment. More simply put, erasing even a single bit of information—such as resetting a computer memory from “1” to “0”—requires a small amount of energy. This principle establishes a fundamental link between information processing and thermodynamics, with the minimum energy required to erase one bit of information at temperature T given by kTln2, where k is the Boltzmann constant (Landauer, 1961).

To refine our model, we incorporate Landauer’s Principle, which states that erasing or resetting one bit of information requires a minimum amount of energy:


(3) $$E_{\min}=\mathrm{kT}\ln2$$

Where:

- $E_{\min}:$ is the minimum energy required to erase one bit (joules)
- k: is Boltzmann’s constant (≈ 1.38 × 10^-23 joules/kelvin)
- T: is the absolute temperature of the system (kelvin)

To incorporate Landauer’s Principle (Equation 3) into the theoretical framework, we need to consider the minimum energy required to process information in the brain. We’ll modify the efficiency factor (η) to account for this minimum energy requirement.

Where:

- t′: Perceptual time (seconds)
- t: Inertial time (seconds)
- k: Boltzmann’s constant (≈1.38 × 10^-23 joules/kelvin)
- T: Absolute temperature of the brain (kelvin)
- η: Efficiency factor (bits/joule)
- I: Total information processed by the brain (bits)
- N: Number of processors (e.g., neurons or functional computing units)
- P: Power consumption per processor (watts)
- R: Brain’s total computational rate (bits/s)
- $I_{f}$: Information processing rate (bits/s)

Step 1: Determine the minimum power required to process information.


(4) $$P_{\min}=E_{\min}\times I_{f}$$

Step 2: Modify the efficiency factor (η) to account for the minimum power requirement (Equation 4).


(5) $$\eta \prime =\eta \times (\frac{P}{P+P_{\min}})$$

Step 3: Express the total information processed (I) in terms of the information processing rate ($I_{f}$) and inertial time (t).


(6) $$I=I_{f}\times t$$

Step 4: Substitute Equations 5, 6 into Equation 2 for perceptual time (t′).


(7) $$t\prime =\frac{I_{f}\times t}{N\times P\times \eta \prime }$$

Step 5: Simplify the Equation 7 by expanding Equation 5.


(8) $$t\prime =\frac{I_{f}\times t}{N\times P\times (\eta \times (\frac{P}{P+P_{\min}}))}$$

Condensing Equation 8 we get the final equation:


(9) $$t\prime =\frac{I_{f}\times t\times (P+(\mathrm{kT}\ln2)\times I_{f})}{N\times P^{2}\times \eta }$$

Units simplify to seconds, confirming dimensional consistency (see Appendix A).

#### 3.5.1 Interpretation

The incorporation of Landauer’s Principle into our theoretical framework offers valuable insights into the relationship between perceptual time and the brain’s energy requirements for information processing. The final equation reveals two critical implications.

First, the inclusion of Landauer’s Principle introduces a term (kTln2) that accounts for the minimum energy required to process information. This term highlights the fundamental physical limit on the brain’s computational efficiency. It suggests that even in an ideal scenario, the brain must consume a certain amount of energy to process and erase information, which may influence perceptual time.

Second, the minimum energy term (kTln2) is directly proportional to the absolute temperature of the brain (T). This implies that as the brain’s temperature increases, the minimum energy required for information processing also increases. Consequently, higher brain temperatures may lead to a dilation of perceptual time. This relationship suggests that variations in brain temperature, whether due to physiological factors or external influences, could modulate our perception of time. A study by Hancock (1993) directly investigated the relationship between body temperature and time perception. The research found that changes in body temperature, particularly increases, led to nonlinear decreases in estimated duration. This finding supports the theory that higher temperatures can lead to a compression in perceptual time, such as during exercise.This topic was explored in a review by Behm and Carter but is lacking comprehensive investigation (Behm and Carter, 2020).

### 3.6 Incorporating Bremermann’s Limit

Bremermann’s Limit, proposed by Bremermann (1962), represents the maximum computational speed of a self-contained system in the material universe. This limit is derived from the mass-energy equivalence and Heisenberg’s Uncertainty Principle, and it sets an upper bound on the rate at which information can be processed per unit of energy (Bremermann, 1962).

Bremermann’s Limit is defined as:


(10) $$R_{\max}=\frac{2\times E_{\text{total}}}{\pi \times \hbar }$$

Where:

- $R_{\max}:$Maximum information processing rate (bits/s)
- $E_{\text{total}}$: Total energy available for computation (joules)
- ℏ: Reduced Planck’s constant (1.05 × 10^-34 joule-seconds)

To account for the physical limitations on the brain’s computational speed, we incorporate Bremermann’s Limit (Equation 10) into the theoretical framework. By constraining the efficiency factor (η) using this limit, we ensure that the model adheres to the fundamental principles governing the maximum rate of information processing per unit of energy.

Step 1: Define the total power consumption of the system as:


(11) $$P_{\text{total}}=N\times P$$

Equation 11 is power which is equivalent to energy per unit time (watts = joules/s), the total energy consumed over a time interval (Δt) is:


(12) $$E_{\text{total}}=P_{\text{total}}\times \mathrm{\Delta t}=N\times P\times \mathrm{\Delta t}$$

However, Bremermann’s Limit concerns the maximum computational rate at any instant, so in Equation 12 we’ll consider (Δt=1s) second for simplicity:


(13) $$E_{\text{total}}=N\times P\times 1=N\times P$$

Step 2: Apply Bremermann’s Limit.

Substitute Equation 13 into Bremermann’s Limit:


(14) $$R_{\max}=\frac{2\times N\times P}{\pi \times \hbar }$$

Equation 14 represents the maximum computational rate achievable by the system based on its total energy consumption.

Step 3: Constrain the efficiency factor (η).

From Equation 2:


(15) R=N×P×η

According to Bremermann’s Limit, (R) must satisfy:


(16) $$R\leq R_{\max}$$

Substitute the expressions for (R) (Equation 15) and (R max) (Equation 14) into Equation 16:


(17) $$N\times P\times \eta \leq \frac{2\times N\times P}{\pi \times \hbar }$$

Simplify both sides of Equation 17 (N×P) (assuming N×P≠0):


(18) $$\eta \leq \frac{2}{\pi \times \hbar }$$

Step 4: Update the Perceptual Time Equation (Equation 2).

The original expression for perceptual time is:


(2) $$t\prime =\frac{I}{N\times P\times \eta }$$

With the constraint on Equation 18, the minimum perceptual time ($t_{\min}^{\prime }$) is achieved when (η) is at its maximum value:


(19) $$\eta_{\max}=\frac{2}{\pi \times \hbar }$$

Substituting Equation 19 into Equation 2:


(20) $$t_{\min}^{\prime }=\frac{I}{N\times P\times \eta_{\max}}$$

Units simplify to seconds, confirming dimensional consistency. (see Appendix A).

#### 3.6.1 Interpretation

The proposed model incorporates Bremermann’s Limit, which introduces a fundamental constraint on the efficiency of information processing in the brain. This limit, derived from quantum mechanical principles, has significant implications for understanding the physical bounds of time perception.

As shown in the updated model (Equation 20), the efficiency factor (η) is subject to an absolute upper limit:

$$\eta_{\max}=\frac{2}{\pi \times \hbar }$$

Where ℏ is the reduced Planck’s constant. This maximum efficiency is determined by fundamental physical constants and represents the theoretical ceiling on how efficiently the brain can convert energy into information processing. The inclusion of this limit in the model highlights a key link between the quantum world and the neurophysiological processes that underlie time perception.

A key consequence of the bounded efficiency factor is the existence of a lower limit to perceptual time (t′) for a given amount of information (I). This implies that the brain cannot perceive or process information instantaneously; instead, there is a minimum time required, dictated by the fundamental laws of quantum mechanics. This insight sheds new light on the temporal resolution of human cognition and suggests that our subjective experience of time is ultimately constrained by the fundamental physical laws that govern reality.

By integrating Bremermann’s Limit into the theoretical framework, we have established a fundamental quantum physical constraint on the brain’s information processing capabilities. This constraint affects the perception of time by introducing a minimum perceptual time required to process a given amount of information. The updated model now accounts for the ultimate efficiency limits imposed by quantum mechanics, providing deeper insights into how physical laws shape cognitive processes.

### 3.7 The combined model: integrating Landauer’s Principle and Bremermann’s Limit

Given:

- t′: Perceptual time (seconds)
- t: Inertial time (seconds)
- k: Boltzmann’s constant (≈1.380649 × 10^-23 joule/kelvin)
- T: Absolute temperature of the brain (kelvin)
- η: Efficiency factor (bits/joule)
- I: Total information processed by the brain (bits)
- N: Number of processors (e.g., neurons or functional computing units)
- P: Power consumption per processor (watts)
- R: Brain’s total computational rate (bits/s)
- $I_{f}$: Information processing rate (bits/s)
- ℏ: Reduced Planck’s constant (1.0545718 × 10^-34 joule-seconds)

Step 1: Substitute Equation 19 into Equation 9:


(21) $$t\prime =\frac{I_{f}\times t\times (P+\mathrm{kT}\ln2\times I_{f})}{N\times P^{2}\times (\frac{2}{\pi \times \hbar })}$$

Step 2: Simplify Equation 21:


(22) $$t\prime =\frac{I_{f}\times t\times (P+\mathrm{kT}\ln2\times I_{f})\times \pi \times \hbar }{2\times N\times P^{2}}$$

Reduce Equation 22 to get the final comprehensive equation:


(23) $$t\prime =\frac{\pi \times \hbar \times I_{f}\times t\times (P+\mathrm{kT}\ln2\times I_{f})}{2\times N\times P^{2}}$$

Units simplify to seconds, confirming dimensional consistency (see Appendix A).

#### 3.7.1 Interpretation

This final equation integrates both Landauer’s Principle and Bremermann’s Limit into the original model, providing a comprehensive understanding of how physical limits affect perceptual time. Equation 23 further suggests that perceptual time is a function of inertial time, modulated by the brain’s information processing rate, power consumption, and efficiency; all of which are constrained by the fundamental physical limits of thermodynamics and quantum mechanics.

The proposed model has several important implications for understanding the nature of time perception. First, it suggests that higher information processing rates ($I_{f}$) lead to an increase in the speed of perceptual time relative to inertial time. This is consistent with the subjective experience of time “flying” when one is actively engaged in demanding mental tasks. Second, the model indicates that higher power consumption (P) and a greater number of processors (N) contribute to a deceleration of perceptual time, which aligns with the notion that non-metabolically expensive neural activity, can make time feel as though it is passing slowly.

While metabolic demand and information throughput usually rise together, perceptual time depends on how efficiently that energy becomes useful information. When higher power per processor (P) is coupled with equal-or-better conversion efficiency (η), throughput R = N ×P× η increases and perceptual time compresses. Conversely, if additional energy is dissipated as overhead (η falls), total power can rise without a proportional gain in useful throughput, shortening perceived duration. The model therefore distinguishes between raw energy expenditure and informational work.

Importantly, the inclusion of Landauer’s Principle and Bremermann’s Limit in the model underscores the idea that even the brain’s perception of time is ultimately constrained by the immutable laws of physics. This highlights the deep connections between the physical world and our perception of it. Additionally, it suggests that a complete understanding of consciousness and perceptual experience must take into account the fundamental physical limitations imposed by the universe. Moreover, the integration with Landauer’s Principle and Bremmerman’s Limit demonstrates that the Perceptual Model of Time is compatible conceptually and mathematically with existing well established physical models of the world.

## 4 Discussion

### 4.1 Implications of the basic theoretical model

The proposed theoretical model, which integrates Landauer’s Principle and Bremermann’s Limit, provides a novel perspective on how the brain’s computational limitations may lead to perceptual time distortions. By abstracting the complexities of neural computations and focusing on key parameters governing information processing capacity, this model offers valuable insights into the fundamental physical constraints that shape our perception of time.

The basic model, as described by Equation 2, establishes a direct proportionality between perceptual time (t′) and the total information (I) processed by the brain:

$$t\prime =\frac{I}{N\times P\times \eta }$$

where (N) denotes the number of parallel processors, (P) represents the power consumption per processor, and (η) signifies the computational efficiency. This relationship implies that an increase in the cognitive load, manifested as a higher (I) can lead to a dilation of perceptual time within a given inertial time interval. This phenomenon aligns with empirical observations where heightened cognitive engagement results in the underestimation of time duration, as individuals allocate more attentional resources to processing information, thereby reducing their temporal awareness (Castellotti et al., 2022).

Furthermore, the model highlights an inverse relationship between perceptual time with respect to both the number of processors (N) and the efficiency (η). An increase in (N) facilitates parallel processing, effectively distributing the cognitive load and potentially leading to a compression of perceptual time. This is corroborated by neuroimaging studies demonstrating that parallel processing networks in the brain contribute to more efficient task performance and altered time perception (Sigman and Dehaene, 2008). Similarly, higher computational efficiency (η) implies that the brain requires less energy to process a given amount of information, which will result in a compression of perceptual time.

### 4.2 Integration of Landauer’s Principle

Incorporating Landauer’s Principle introduces a thermodynamic constraint on the minimum energy ($E_{\min}$) required for information processing:

$$E_{\min}=\mathrm{kT}\ln2$$

where (k) is the Boltzmann constant and (T) is the absolute temperature of the brain. By embedding this principle into the model, we recognize that energy consumption is not merely a biological parameter but is fundamentally limited by thermodynamics. The modified equation (Equation 9) accounts for the energy-temperature dependence, suggesting that increases in brain temperature (T) raise the minimum energy threshold ($E_{\min}$), potentially leading to a dilation of perceptual time. This theoretical insight is supported by experimental findings indicating that elevated body temperatures can accelerate the subjective passage of time (Hancock, 1993; Behm and Carter, 2020).

### 4.3 Incorporation of Bremermann’s Limit

Bremermann’s Limit imposes a quantum mechanical ceiling on the rate of information processing, constraining the maximum computational efficiency $(\eta_{\max}):$

$$\eta_{\max}=\frac{2}{\pi \times \hbar }$$

where (ℏ) is the reduced Planck constant.

This quantum constraint introduces a lower limit to temporal resolution in cognitive processes, suggesting that our subjective perceptual experience of time is inherently quantized and bounded by the universe’s fundamental properties (Buice and Cowan, 2009; Atmanspacher and Filk, 2002). In turn, this limit underscores that there is an absolute bound on how efficiently energy can be converted into information processing, dictated by fundamental physical constants. The implication is profound: there is a minimum perceptual time ($t_{\min}^{\prime }$) necessary for the brain to process a given amount of information (I), precluding the possibility of instantaneous perception (Bruza et al., 2009).

### 4.4 The combined model and its significance

By synthesizing the basic model with both Landauer’s Principle and Bremermann’s Limit, we arrive at a comprehensive equation that captures the complex dependencies of perceptual time:


$$t\prime =\frac{\pi \times \hbar \times I_{f}\times t(P+\mathrm{kT}\ln2\times I_{f})}{2\times N\times P^{2}}$$

This final expression emphasizes that perceptual time is a function of inertial time, modulated by the brain’s information processing rate, power consumption, and constrained by both thermodynamic and quantum mechanical limits.

The integration of fundamental physical principles into the model not only bridges the gap between psychology, neurophysiology and physics but also highlights that cognitive processes are ultimately governed by universal laws. The recognition that perceptual time is constrained by Landauer’s Principle and Bremermann’s Limit provides a novel perspective on the limitations of the human brain.

### 4.5 Relation to existing literature

Our theoretical findings resonate with and extend existing research on time perception and cognitive load. Studies have consistently shown that increased cognitive demands can distort time perception, leading to either an underestimation or overestimation of elapsed time (Block et al., 2010). The incorporation of thermodynamic and quantum constraints offers a foundational explanation for these phenomena, rooted in the physical limitations of information processing.

Moreover, the model’s implication that temperature influences perceptual time is supported by experimental work demonstrating that physiological states affecting body temperature can alter time perception (Wearden et al., 2007). By framing these observations within the context of Landauer’s Principle, we provide a thermodynamically grounded rationale for such effects.

### 4.6 Implications for time perception in AI systems

Building on this capacity-limited framework, one can draw parallels between biological time perception and the emergent “perceptual time” within artificial intelligence (AI) systems. Just as the brain’s finite information-processing capabilities constrain the subjective flow of time (Panagiotaropoulos et al., 2012; Dormal et al., 2012), advanced AI models configured with hardware, energy budgets, and algorithmic constraints must also operate within strict computational limits. The result is that AI “experiences” a form of time not solely governed by an external clock but by the interplay of processing throughput and energy considerations, akin to the rate-limiting factors in human neural tissue (Simen et al., 2013; Addyman et al., 2011). Though the notion of AI consciousness remains a topic of debate and investigation, these capacity-driven constraints imply that any sufficiently complex system might develop an emergent internal chronometry that parallels human perceptual time (Basgol et al., 2021). In this sense, AI becomes “aware” of temporal progression insofar as it encodes and updates internal states according to a rate of information processing that is fundamentally constrained by power consumption, efficiency, and the number of parallel processing units. Thus, whether in cortical networks or digital architectures, perceptual time can be viewed as an adaptive byproduct of computational limits, providing a unified theoretical perspective on how systems biological or artificial come to register, track, and even subjectively experience the flow of time.

Unlike biological observers, current AI architectures process time exclusively through deterministic clock cycles and algorithmic scheduling; they lack the affective states and memory distortions that generate human temporal illusions. Consequently, any divergence between ‘perceptual’ and inertial time in today’s AI is not phenomenological but purely computational (e.g., variable task-queue latency or throttled throughput). Whether future systems develop a genuinely *subjective* temporality and thus exhibit a true perceptual inertial gap, remains an open empirical question.

### 4.7 Implications for brain computer interface systems

As we begin to integrate neurotechnology more deeply the concept of perceptual time becomes increasingly critical. By directly interfacing with neural pathways, BCIs could augment or even override the brain’s intrinsic temporal dynamics, opening up the possibility of effectively hyper compressing or “freezing” inertial time for the user. In principle, if the processing and resource constraints are optimized correctly, one could create intervals of experience where large amounts of cognitive operations occur while external time appears to stand still.

### 4.8 Implications for the finite range of human computation

The proposed theoretical model, which establishes fundamental physical limits on the brain’s computational capacity, raises intriguing questions about the existence of a finite range of computation within which all humans operate. If the brain’s information processing is indeed constrained by thermodynamic and quantum mechanical bounds, as suggested by the integration of Landauer’s Principle and Bremermann’s Limit, it implies that there may be a universal “cognitive envelope” that defines the boundaries of all human thought and perception. Further research and development of the model may elucidate other fundamental physical characteristics of the universe bounded by human perception rather than traditional physics.

### 4.9 Limitations and future directions

While the model offers significant insights, it remains a simplification of the complex neural processes underlying time perception. The brain’s information processing is modulated by a myriad factors, including attention, emotional state, memory, and sensory inputs, which are not explicitly captured in the current framework. Additionally, parameters such as the number of processors (N) and efficiency (η) are treated abstractly, without precise quantification in neurobiological terms.

Future research should aim to empirically validate the model’s predictions by quantifying these parameters using neuroimaging and neurophysiological techniques. Investigations into how variations in brain temperature, metabolic rates, and neural efficiency affect time perception could further refine the model. Incorporating stochastic elements to account for the probabilistic nature of quantum mechanics may also enhance the model’s applicability to real-world cognitive processes.

## 5 Proposed methodology to assess computational load and subjective time

This methodology provides a means to assess whether the brain’s “computational energy”—inferred through EEG measures such as power spectral density (PSD), event-related desynchronization (ERD), and global field power (GFP)—correlates with shifts in perceived time. By contrasting baseline resting-state EEG data with recordings obtained during low-and high-load cognitive tasks, researchers can estimate the degree of “extra” neural activity or energy the brain expends under more demanding conditions. These EEG-based indices serve as proxies for the overall information-processing rate (*R*), which, directly influences perceptual time (*t′*). Participants then estimate or reproduce the duration of each task, allowing for a comparison between actual clock time (*t*) and perceived time (*t′*). If the data reveals that higher EEG-derived energy correlates with a greater discrepancy in time judgments, it would suggest that computational load meaningfully contributes to time dilation or compression—thereby supporting the idea that (*t′*​) scales with changes in information-processing in the brain.

To systematically manipulate the brain’s *total* computational capacity (*R*), participants first complete maximal-performance baseline tests (linguistic/mathematical) establishing peak cognitive throughput. Following overnight sleep deprivation—a physiologically validated method for degrading neurocognitive capacity (Durmer and Dinges, 2005)—subjects repeat identical testing protocols. This fatigue-conditioned replication introduces controlled variation in global computational resources, complementing task-evoked (*R*) modulations. The resultant dataset (rested/high-load, rested/low-load, fatigued/high-load, fatigued/low-load) enables differentiation between transient neural energy expenditure and sustained capacity constraints in temporal perception.

Such outcomes would indicate that the underlying neural mechanisms responsible for advanced cognitive processing might transiently distort time perception, in line with the hypothesis that perceptual time depends on both total information processed (*I*) and the brain’s total capacity to process it (*R*). Conversely, a weak or null correlation would imply that factors beyond energy expenditure—such as attention, arousal, or emotional state—may play a larger role in shaping temporal judgments. Further research might refine this approach by integrating more direct measures of metabolic consumption (e.g., fMRI or PET) to confirm how closely EEG-based “energy” metrics track actual cortical resource usage. Ultimately, combining task paradigms of varying complexity with multimodal imaging could clarify not only how computational load influences perceptual time but also whether individual differences in efficiency (η) mitigate or amplify subjective distortions.

### 5.1 Parameter interpretability and neural correlates

To facilitate furtherempirical validation and integrate our model with existing neuroscientific literature, we propose potential neurobiological correlates for each parameter:

**Table 1:** 

Parameter	Neurobiological correlate
I(Information Processed)	Mutual information, spike entropy, synaptic throughput
N(Processors)	Number of concurrently active neural ensembles (via EEG/fMRI coherence)
P(Power Consumption)	Cerebral metabolic rate (e.g., fMRI BOLD, PET glucose uptake)
η (Efficiency)	Bits per joule; derived from entropy-to-energy ratios

This mapping allows for direct empirical testing of the model. For example, researchers could manipulate cognitive load while measuring both neural activation and subjective time estimates. According to our model, changes in activation should correlate with distortions in perceived duration proportional to the ratio between information processed (I) and computational capacity (R).

Our model’s emphasis on temporal processing networks aligns with the “time-domain brain” framework recently proposed by Cariani and Baker (2022), which posits that the brain’s signals themselves are fundamentally composed of temporal patterns. Their framework highlights how neural spike timing serves as the raw material for information coding, describing multiple temporal representation strategies including linear, cyclical, ordinal, wave, and anticipatory time processing. This perspective complements our model by providing neurobiological mechanisms through which parameters like information processing rate (R) might be implemented. Specifically, Baker and Cariani’s emphasis on phase-locking as a “workhorse of temporal coding” and their description of neural integration windows defining temporal coincidence offers potential neural substrates for our efficiency parameter (η). Their work on temporal coherence supporting large-scale coordination further illuminates how our parameter (N) might be realized through distributed neural assemblies that maintain precise temporal relationships. Incorporating this time-domain perspective strengthens the neurobiological plausibility of our computational model and suggests additional EEG measures such as phase synchrony and cross-frequency coupling—that could serve as physiological correlates of the Perceptual Model of Time.

Building on Baker and Cariani’s (2025) “time-domain brain” framework, which formalises how time-delay networks, nested oscillatory cascades, and correlation-based wave-interference (holographic-like) processes cooperate to yield distributed, content-addressable codes. Another potential mapping of their mechanisms onto our parameters is as follows: information throughput (I) scales with the density and multiplexing of spike-time patterns; parallel processing power (N) is realised by ensembles of delay-tuned coincidence detectors whose phase-aligned outputs mutually reinforce; and energetic efficiency (η) depends on constructive interference, minimal destructive cancellation, and the metabolic savings of multiplexing. Baker & Cariani identify measurable meso-scale markers—high-order phase synchrony, cross-frequency phase-amplitude coupling, and potentially rapid microstate transitions—whose presence or absence can test whether perceptual-time dilation or compression tracks not only gross power changes but also the emergence of new carrier-mixing products and sharpened delay-specific phase relations. Thus the time-domain framework supplies concrete biophysical substrates and testable signal-level indices for every parameter of our perceptual-time model, deepening its explanatory reach and empirical falsifiability.

A complementary theoretical perspective worth considering is the controversial holographic proposal advanced by Fields et al. (2021), which conceptualizes neurons as quantum reference frames (QRFs) within an active-inference free-energy framework. This approach posits that neural ensembles implement holographic encoding wherein information exchange across a “holographic screen” between observer and environment is fundamentally classical, despite underlying quantum processes. Within this paradigm, our parameter number of processors (N) could be reinterpreted as the dimensionality of available QRFs that define measurement contexts, while efficiency (η) might reflect the thermodynamic costs of maintaining stable reference frames—costs that Fields et al. demonstrate scale with ln2NkBT. Their formalism suggests that temporal coherence emerges from phase-aligned QRFs whose stability requires energy expenditure proportional to information stored, offering a potential explanation for why higher computational loads (increased I) might distort perceptual time. Although speculative, this perspective aligns with our model by suggesting that perceptual time compression may arise when neural systems deploy additional reference frames to process complex information, thereby breaking symmetries in the underlying quantum description and inducing measurable changes in EEG coherence patterns. Future work could test this hypothesis by examining whether tasks that distort perceptual time also produce the specific cross-frequency coupling signatures predicted when additional QRFs are recruited during cognitive processing.

Moreover, theoretical work by Fields et al. (2022) reframes each neuron as a hierarchy of QRFs that must be actively stabilised “at Landauer-limited energetic cost” to support Bayesian model selection over its local micro-environment. Within this holographic, active-inference picture, the *rate at which QRFs are written, erased, and refreshed* sets an upper bound on the neuron’s information-throughput and, by extension, on the brain-wide processing rate (R) that is fundamental to our Perceptual Model of Time. Crucially, the maintenance of multiple, mutually-compatible QRFs is predicted to (i) elevate electrophysiological power in the beta–gamma range, (ii) shorten effective neural integration windows as decoherence accelerates, and (iii) exaggerate subjective time dilation when tasks demand finer system identification. By augmenting our EEG protocol with microstate-transition density, phase-slip counts, and fronto-parietal permutation entropy we can test whether spikes in “computational energy” reflect the hidden cost of sustaining these frames. A positive correlation between QRF-sensitive metrics, elevated R, and expanded estimates of elapsed duration would therefore provide convergent evidence that perceptual time is co-determined by both classical metabolic load *and* the quantum-informational bookkeeping required to keep the nervous system’s internal coordinate systems coherent.

## 6 Public significance

This study introduces a novel theoretical framework for understanding how the brain’s information processing capacity shapes our perception of time. By distinguishing “perceptual time” from inertial clock time, the model explains phenomena like time dilation and compression during intense experiences as outcomes of neural computational limits. These insights not only enhance our understanding of human cognition but also have implications for artificial intelligence systems and brain-computer interfaces, potentially paving the way for technologies that could manipulate or augment our experience of time.

## Data Availability

The original contributions presented in the study are included in the article/Supplementary material, further inquiries can be directed to the corresponding author.
