# Supplementary material for: Super special relativity
Source: Front Comput Neurosci. 2025 Aug 13;19:1597914. doi: 10.3389/fncom.2025.1597914 (PMC12380679; doi:10.3389/fncom.2025.1597914)
Supplement: Supplementary file 1 [file Supplementary_file_1.docx]

**APPENDIX A**

**3.4 Incorporating Landauer's Principle**

Step 1: Determine the minimum power required to process information.

P_min = E_min × I_f

P_min = (k T ln 2) × (bits/second)

P_min = (Joules/bit) × (bits/second)

P_min = Joules/second = Watts

Step 2: Modify the efficiency factor η to account for the minimum power requirement.

η' = η × (P / (P + P_min))

η' = (bits/Joule) × (Watts / Watts)

η' = bits/Joule

Step 3: Update the equation for perceptual time t' using the modified efficiency factor η'.

t' = I / (N × P × η')

t' = bits / (N × Watts × (bits/Joule))

t' = bits / (N × (Joules/second) × (bits/Joule))

t' = bits / (N × bits/second)

t' = seconds

**3.5 Incorporating Bremermann's Limit**

Step 1: Determine the maximum information processing rate E_max using Bremermann's limit.

E_max = 2E / (π ℏ)

E_max = 2 × (N × P × η) / (π × (Joule seconds))

E_max = 2 × (N × (Joules/second) × (bits/Joule)) / (π × (Joule seconds))

E_max = (2 × N × bits) / (π × seconds)

E_max = bits/second

Step 2: Modify the equation for perceptual time to account for the maximum information processing rate E_max.

t' = I / min(E, E_max)

t' = bits / min((N × bits/second), (bits/second))

t' = bits / (bits/second)

t' = seconds

**3.6 The Combined Model: Integrating Landauer's Principle and Bremermann's Limit**

Step 1: Substitute Equation (20) into Equation (9):

t' = (I_f × t × (P + kT ln(2) × I_f)) / (N × P^2 × η_max)

t' = ((bits/second) × seconds × ((Joules/second) + ((Joules/Kelvin) × Kelvin × (dimensionless) × (bits/second)))) / ((dimensionless) × (Joules/second)^2 × (bits/Joule))

t' = ((bits × Joules) + (bits × Joules)) / (bits/second)

t' = (bits × Joules) / (bits/second)

t' = Joules / (1/second)

t' = Joules × seconds

t' = Joule seconds

t' = (I_f × t × (P + kT ln(2) × I_f)) / (N × P^2 × (2/(π × ℏ)))

t' = ((bits/second) × seconds × ((Joules/second) + ((Joules/Kelvin) × Kelvin × (dimensionless) × (bits/second)))) / ((dimensionless) × (Joules/second)^2 × ((dimensionless) / ((dimensionless) × (Joule seconds))))

t' = ((bits × Joules) + (bits × Joules)) / ((Joules^2/second^2) × (1/(Joule seconds)))

t' = (bits × Joules) / (Joules/second)

t' = bits × seconds

Step 2: Simplify the Equation:

t' = (I_f × t × (P + kT ln(2) × I_f) × π × ℏ) / (2 × N × P^2)

t' = ((bits/second) × seconds × ((Joules/second) + ((Joules/Kelvin) × Kelvin × (dimensionless) × (bits/second))) × (dimensionless) × (Joule seconds)) / ((dimensionless) × (dimensionless) × (Joules/second)^2)

t' = ((bits × Joules) + (bits × Joules)) / (Joules^2/second^2)

t' = (bits × Joules) / (Joules/second)

t' = bits × seconds

Final Comprehensive Equation:

t' = (π ℏ I_f t (P + kT ln(2) × I_f)) / (2NP^2)

t' = ((dimensionless) × (Joule seconds) × (bits/second) × seconds × ((Joules/second) + ((Joules/Kelvin) × Kelvin × (dimensionless) × (bits/second)))) / ((dimensionless) × (dimensionless) × (Joules/second)^2)

t' = (Joule seconds × bits × Joules) / (Joules^2/second)

t' = (Joule^2 × bits × seconds) / (Joules^2)

t' = bits × seconds

(bits are unitless)

t' = seconds

The units are consistent throughout the equations, and the final result for perceptual time (t') is in seconds.
